# Supplementary material for: The NMDA receptor regulates competition of epithelial cells in the Drosophila wing
Source: Nat Commun. 2020 May 6;11:2228. doi: 10.1038/s41467-020-16070-6 (PMC7203100; doi:10.1038/s41467-020-16070-6)
Supplement: Supplementary file 1 — Supplementary Information [file 41467_2020_16070_MOESM1_ESM.pdf]

Supplementary Information

The NMDA receptor regulates competition of epithelial cells

Banreti and Meier, 2020

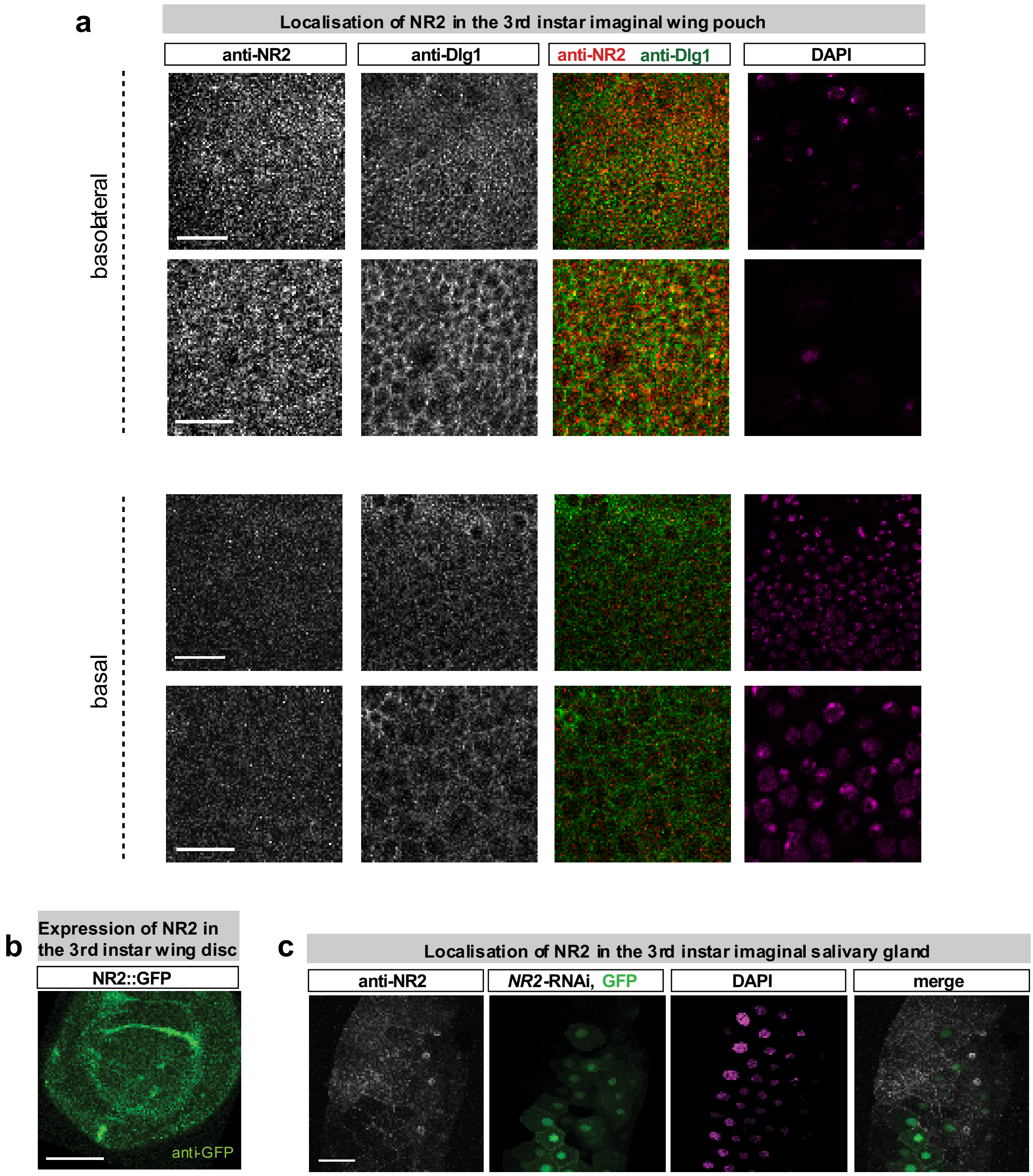

Supplementary Figure 1. Expression of NR2 in wing discs and salivary glands

a, Localisation and expression of NR2 and Dlg1 in the wing disc of third instar larvae was evaluated using the indicated antibodies. Septate junctions are marked with anti-Dlg1. Scale bars 20 µm and 10 µm. **b,** Expression analysis of NR2 in the wing disc of third instar larvae. A protein trap line that carries eGFP in frame with NR2 was used to monitor NR2 expression. Shown is a confocal image of a wing disc stained with anti-GFP (NR2). Scale bar 100 µm. **c,** Expression analysis of NR2 in salivary glands. Immunostaining with anti-NR2 antibodies in salivary glands in which NR2 was downregulated in clones. RNAi clones are marked by GFP. Scale bar 100 µm. Experiments were repeated two independent times. See Supplementary Table 1 for genotypes.

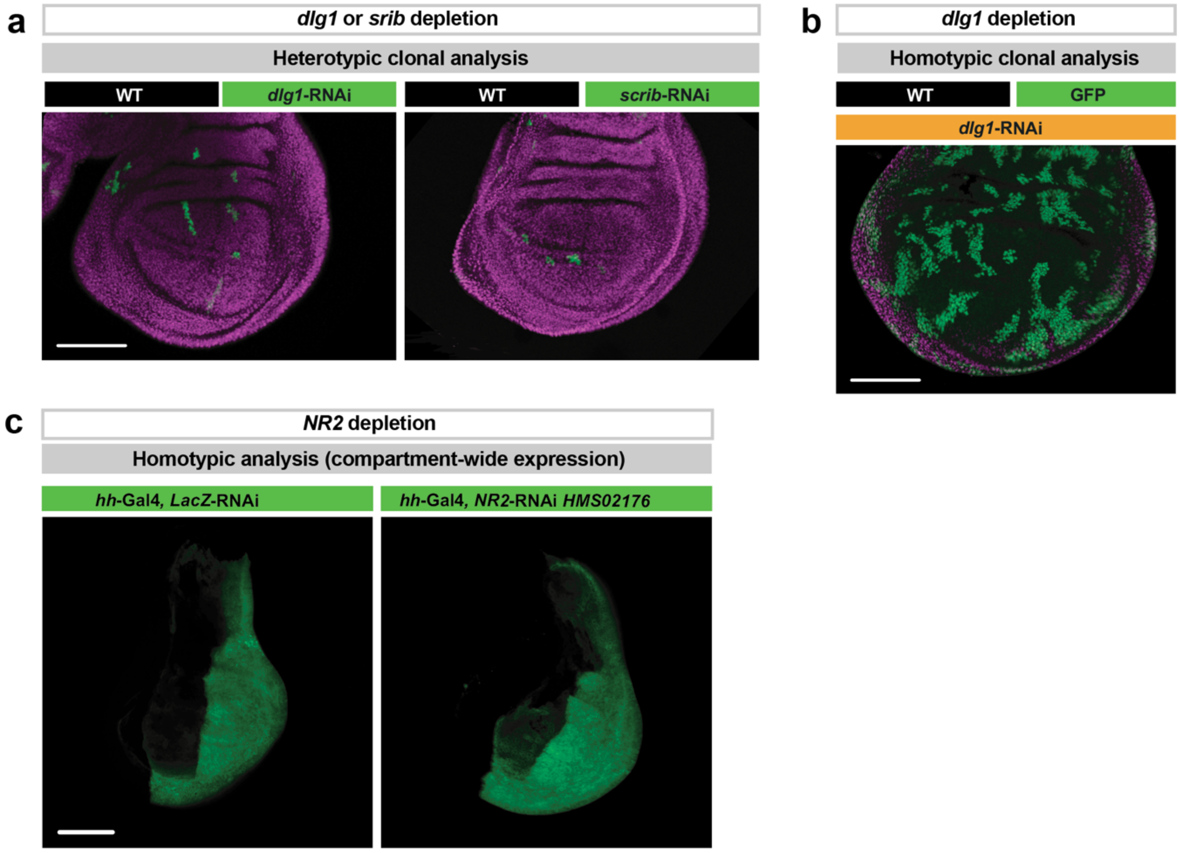

Supplementary Figure 2. Context-dependent elimination of loser clones

a, Heterotypic clonal analysis. The indicated genes were knocked down in *GFP*-marked clones as depicted in Fig. 1d. Scale bar 100 µm. **b,** Homotypic clonal analysis. *dlg1* was knocked down throughout the wing pouch as described in Figure 1f. GFP-marked clones represent pseudo-clones. Scale bar 100 µm. **c,** Homotypic analysis. The indicated genes *were* knocked down throughout the posterior compartment of the wing pouch. Experiments were repeated two independent times. Scale bar 100 µm. See Supplementary Table 1 for genotypes. All experiments, apart from c, were repeated three indepen dent times.

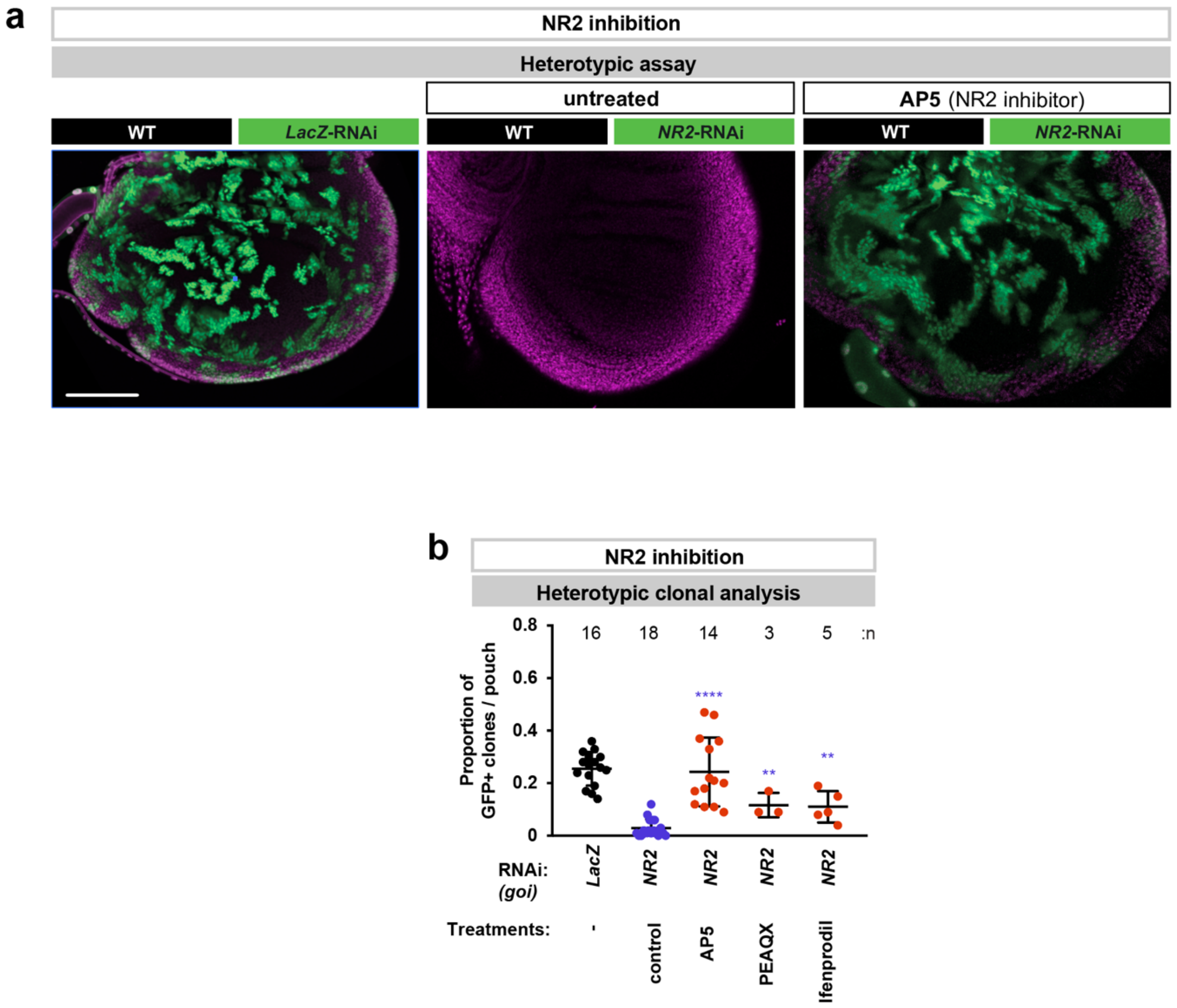

Supplementary Figure 3. Inhibition of NR2 with AP5 antagonist prevents cell competition

a, Heterotypic clonal analysis in the presence or absence of AP5, an inhibitor of NR2. Scale bar 100 µm. Experiments were repeated three independent times. **b,** Quantification of the NR2 agonists treatment assays. Error bars represent average occupancy of the indicated RNAi clones per wing pouch ± SD. ****P<0.0001, ***P<0.001, **P<0.01, *P<0.1 by two-sided Mann-Whitney nonparametric U-test. *(NR2*-RNAi + PEAQX vs. *NR2*-RNAi not treated **P value: 0.0060; *NR2*-RNAi + Ifenprodil vs. *NR2*-RNAi not treated **P value: 0.0028). n depicts the number of wing discs. See Supplementary Table 1 for genoty pes.

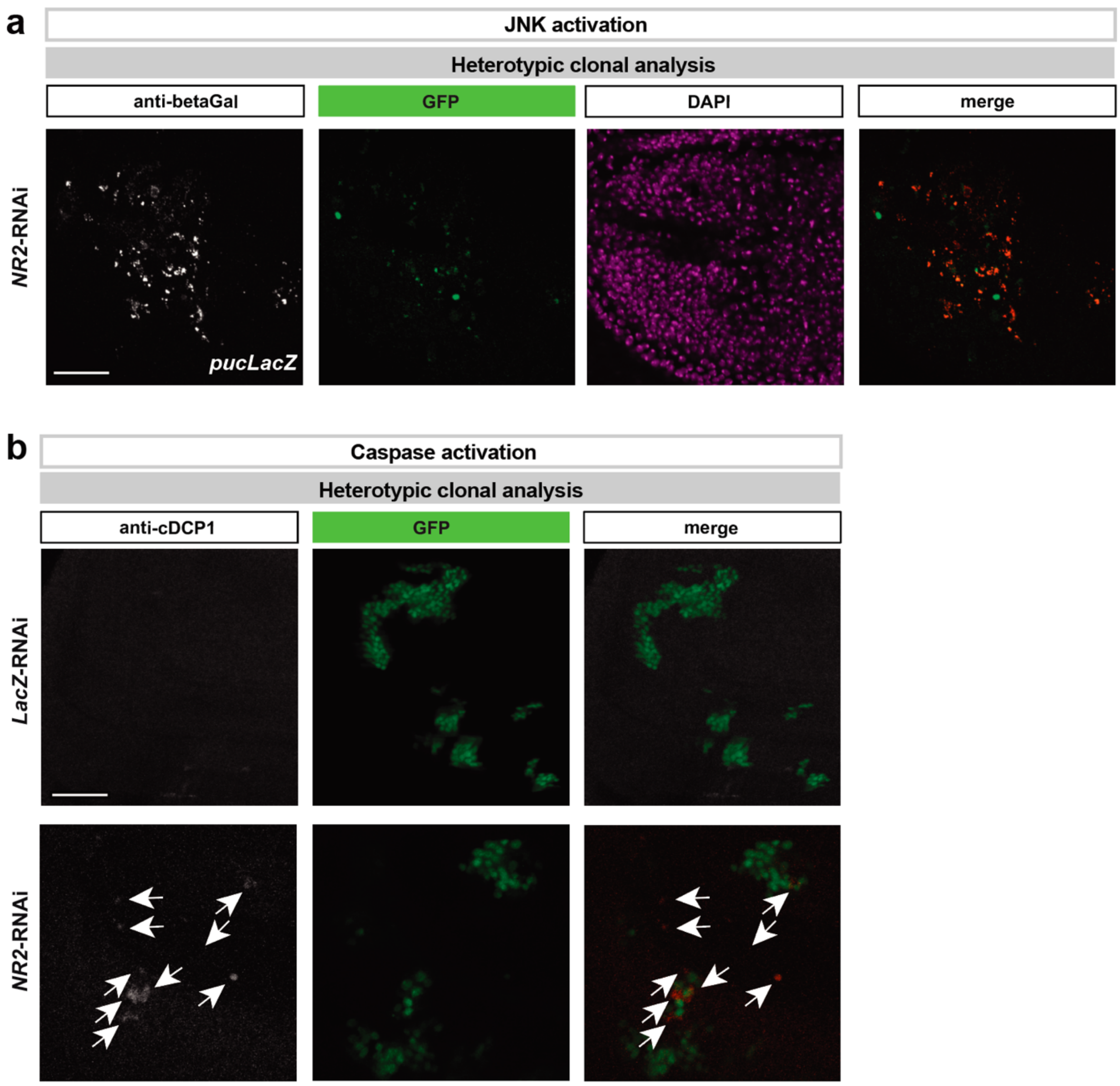

Supplementary Figure 4. *NR2* loser clones are eliminated by apoptosis via the Eiger>JNK signalling axis

a, Heterotypic clonal analysis of third instar wing discs. *PucLacZ*, expression as a marker of *JNK* activity was revealed with anti-*β*Gal staining. Scale bar 50 μm. Experiment was repeated two independent tim es. **b**, Confocal images of wing discs that were immunostained with anti-cleaved DCP1. Scale bar 50 μm. Experiments were repeated three independent times. See Supplementary Table 1 for genotypes.

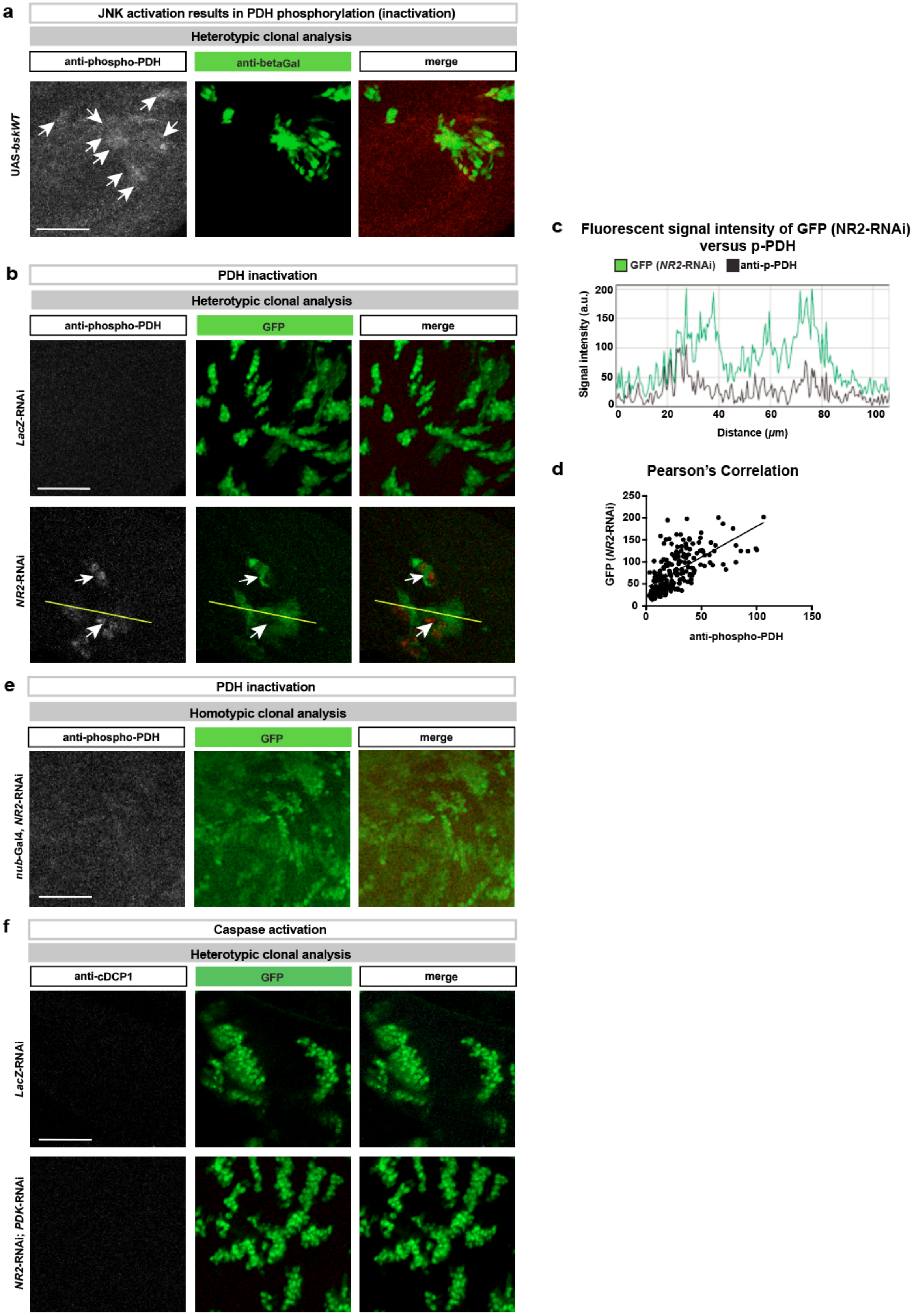

Supplementary Figure 5. NR2 controls cell competition by regulating lactate-mediated metabolic coupling between winners and losers

a-b, Confocal images of anti-βGal (marking *UAS-LacZ* clones) and anti-phospho-PDH (reading out its inactivation) stained wing pouches. Scale bar 50 µm. **c,** Fluorescent intensities of phospho-PDH (black) and GFP (green) are measured by ImageJ software at the yellow line. Experiments were repeated three independent times. **d,** Pearson's correlation analysis of phospho-PDH and GFP. **e,** Homotypic analysis. Confocal images of anti-phospho-PDH stained wing pouches. Scale bar 50 µm. **f,** Confocal images of third instar imaginal wing discs that were immunostained for cleaved DCP1 as a readout of caspase activation and apoptosis. Scale bar 50 µm. Experiments were repeated two independent times, unless stated otherwise. See Supplementary Table 1 for genotypes.

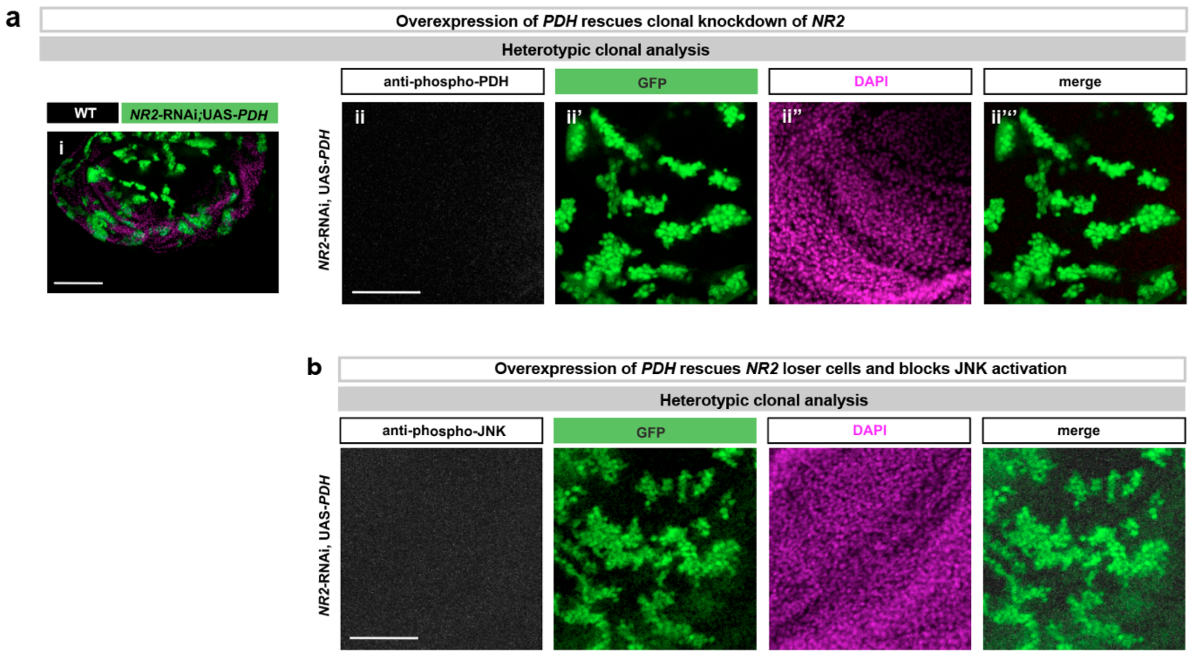

Supplementary Figure 6. The TNF>JNK>PDK>PDH signalling axis reprograms the metabolism of *NR2* loser clones

a, Heterotypic clonal analysis. The indicated gene-of-interest (goi) was over-expressed (*UAS-PDH*) in GFP-marked *NR2*-RNAi clones, and stained for anti-phospho-PDH (ii). Clones are marked by GFP (green). Scale bar 100 µm (i) or 50 µm (ii). **b,** Confocal images of wing discs that were immunostained with anti-phospho-JNK. Scale bar 100 µm. Experiments were repeated three independent times. See Supplementary Table 1 for genotypes.

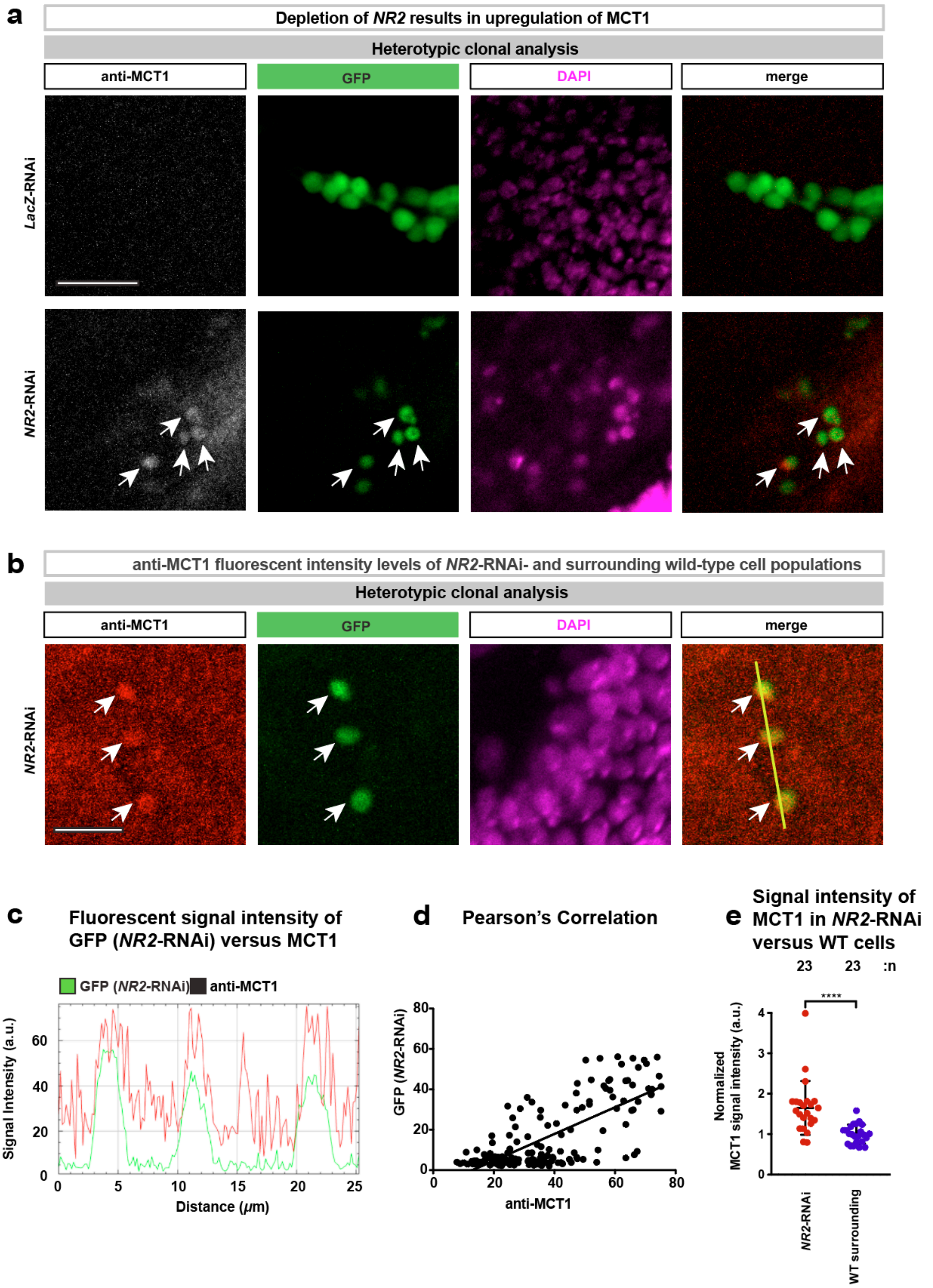

Supplementary Figure 7. MCT1 is upregulated in *NR2* loser clones

a,b, Confocal images of anti-MCT1 stained wing pouches. Scale bars 20 μm (a) and 10 μm (b). See S upplementary Table 1 for genotypes. **c,** Fluorescent intensities of anti-MCT1 (red) and GFP

(green) are measured by ImageJ software at the yellow line. **d,** Pearson's correlation analysis of anti-MCT1 and GFP. **e,** Fluorescent intensities (error bars) of anti-MCT1 are measured by ImageJ software ± SD. ****P<0.0001, ***P<0.001, **P<0.01, *P<0.1 by two-sided Mann-Whitney nonparametric *U*-test. n depicts the number of cells analysed. Experiments were repeated three independent times. See Supplementary Table 1 for genotypes.

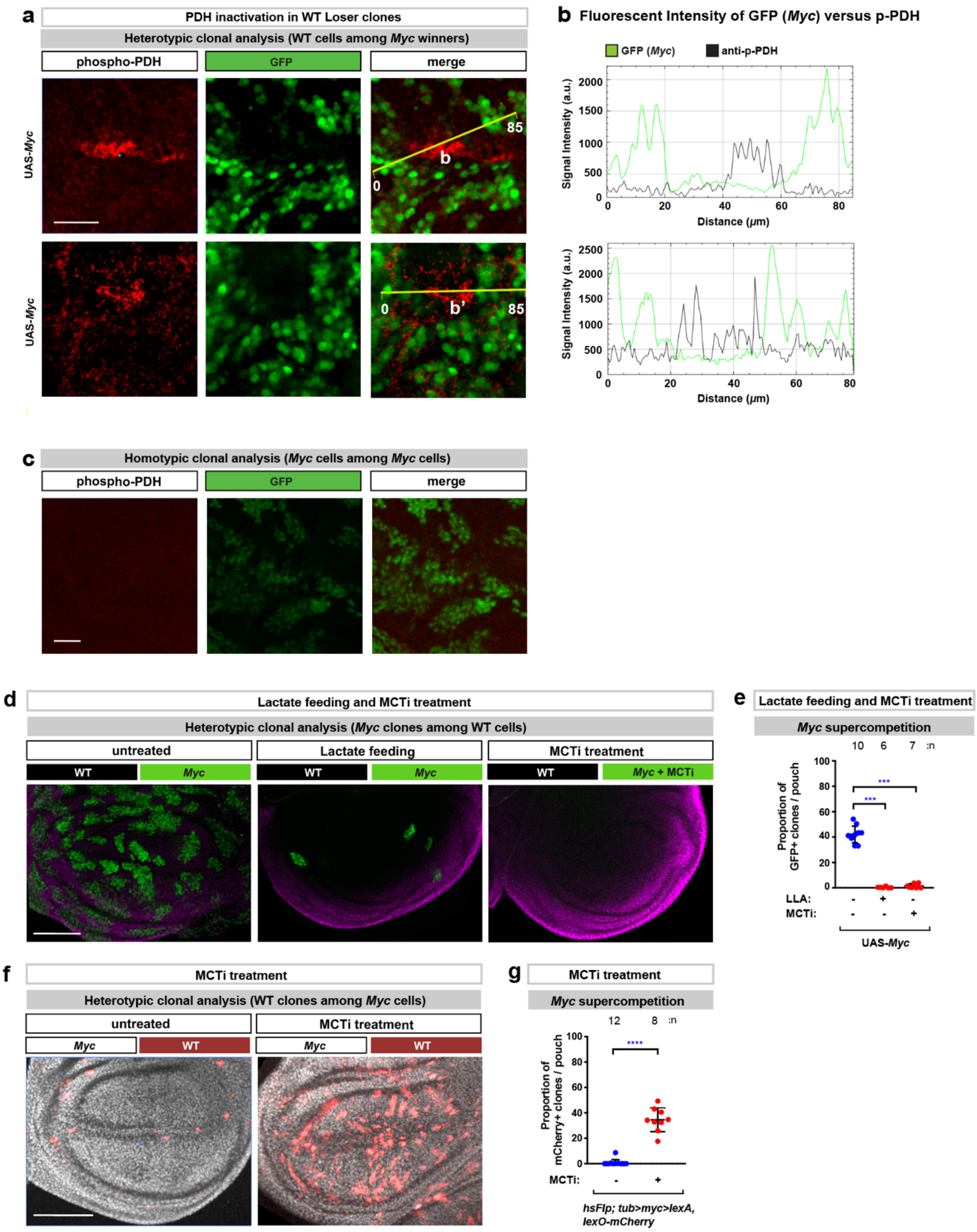

Supplementary Figure 8. Lactate-mediated metabolic coupling occurs during loser/Myc supercompetition

a, Heterotypic clonal analysis. Confocal images of dissected wing pouches stained for anti-phospho-PDH (reading out its inactivation). Scale bar 25 µm. **b,** Fluorescent intensities of phospho-PDH (black) and GFP (green) are measured by ImageJ software at the yellow lines. **c,** Homotypic clonal analysis. Confocal images of dissected wing pouches stained for anti-phospho-PDH (reading out its inactivation). Scale bar 25 µm. **d,** Heterotypic clonal analysis. Myc was over-expressed in *GFP*-marked clones. For

genotypes see Supplementary Data Table. Clones are marked by GFP (green). Lactate feeding and MCTi treatment was conducted as outlined in the Methods section. Scale bar 100 μm **e,** Quantification of the Lactate (LLA) and MCTi treatment assays. **f)** Myc supercompetition assay. WT cells are marked with *mCherry*. Treatment with MCT1 inhibitor was conducted as outlined in the Methods section. Scale bar 100 μm**. g,** Quantification of the MCTi treatment assay. See Supplementary Data Table for genotypes. Error bars represent average occupancy of the indicated UAS clones per wing pouch ± SD. ****P<0.0001, ***P<0.001, **P<0.01, *P<0.1 by two-sided Mann-Whitney nonparametric U-test. (e,: UAS-*Myc* LLA fed vs. UAS-*Myc* not treated ***P value: 0.0002; UAS-*Myc* MCTi treated vs. UAS-*Myc* not treated ***P value: 0.0001). n depicts the number of wing discs. See Supplementary Table 1 for genotypes.

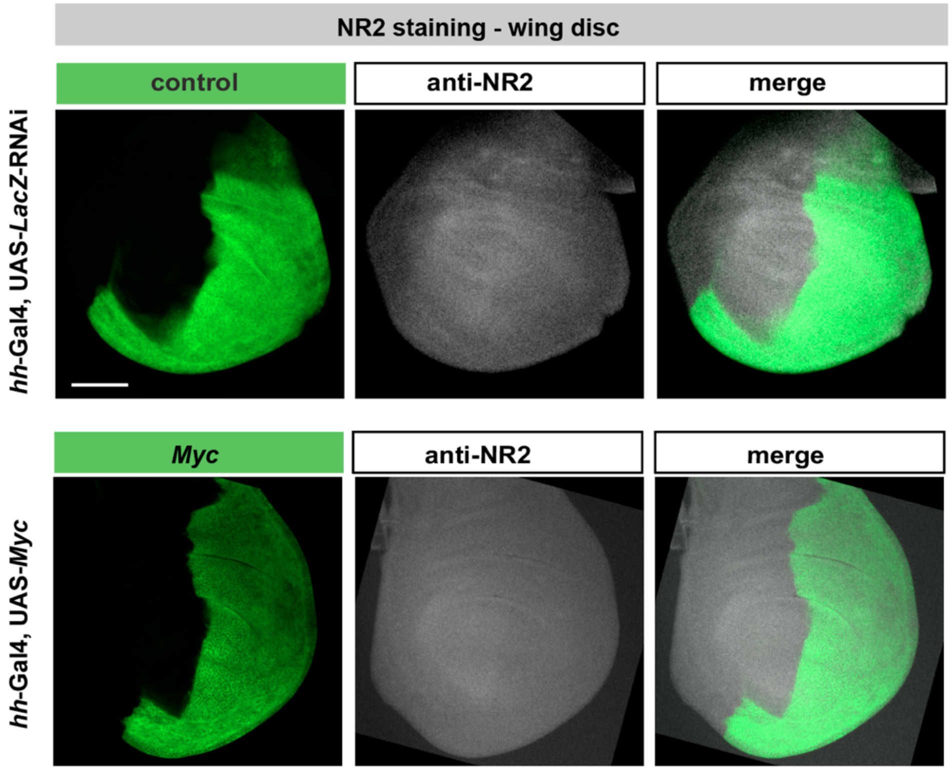

Supplementary Figure 9. NR2 staining in the wing pouch

Antibody staining with anti-NR2 antibodies in wing discs in which Myc was over-expressed throughout the posterior compartment of the wing pouch (marked by GFP). Scale bar 100 μm. See Supplementary Table 1 for genotypes.

Supplementary Table 1. *Drosophila* genotypes used in this study

Main Figures

Figure:	Panel:	Genotype:
1	c	• *hsFlp;UAS-LacZ-RNAi/+;act>CD2>Gal4,UAS-nEGFP/+*<br>• *hsFlp;+/+;act>CD2>Gal4,UAS-nEGFP/*$NR2^{GD3196}$*-RNAi*<br>• *hsFlp;UAS-NR2-RNAi/+;act>CD2>Gal4,UAS-nEGFP/+*
1	d	• *hsFlp;UAS-LacZ-RNAi/+;act>CD2>Gal4,UAS-nEGFP/+*<br>• *hsFlp;+/+;act>CD2>Gal4,UAS-nEGFP/UAS-*$dlg1^{GD41136}$*-RNAi*<br>• *hsFlp;UAS-*$scrib^{TRIP-GL00638}$*-RNAi/+;act>CD2>Gal4,UAS-nEGFP/+*<br>• *hsFlp;+/+;act>CD2>Gal4,UAS-nEGFP/*$NR2^{GD3196}$*-RNAi*<br>• *hsFlp;UAS-*$NR2^{TRIP-HMS02176}$*-RNAi/+;act>CD2>Gal4,UAS-nEGFP/+*<br>• *hsFlp;UAS-NR2-RNAi/+;act>CD2>Gal4,UAS-nEGFP/+*<br>• *hsFlp;+/+;act>CD2>Gal4,UAS-nEGFP/*$NR2^{TRIP-HMS02012}$*-RNAi*<br>• *hsFlp;+/+;act>CD2>Gal4,UAS-nEGFP/*$NR2^{TRIP-JF02044}$*-RNAi*
1	e	• *hsFlp;UAS-LacZ-RNAi/nub-Gal4;ubi-p63E>FRT.STOP.FRT>Stringer(nEGFP)/+*<br>• *hsFlp;+/nub-Gal4;ubi-p63E>FRT.STOP.FRT>Stringer(nEGFP)/*$NR2^{GD3196}$*-RNAi*
1	f	• *hsFlp;UAS-LacZ-RNAi/+;act>CD2>Gal4,UAS-nEGFP/+*<br>• *hsFlp; UAS-LacZ-RNAi/nub-Gal4;ubi-p63E>FRT.STOP.FRT>Stringer(nEGFP)/+*<br>• *hsFlp;+/nub-Gal4;ubi-p63E>FRT.STOP.FRT>Stringer(nEGFP)/UAS-*$dlg1^{GD41136}$*-RNAi*<br>• *hsFlp; UAS-*$NR2^{TRIP-HMS02176}$*-RNAi/nub-Gal4;ubi-p63E>FRT.STOP.FRT>Stringer(nEGFP)/+*<br>• *hsFlp;+/nub-Gal4;ubi-p63E>FRT.STOP.FRT>Stringer(nEGFP)/*$NR2^{GD3196}$*-RNAi*
1	g	• *hsFlp; UAS-LacZ-RNAi/nub-Gal4;ubi-p63E>FRT.STOP.FRT>Stringer(nEGFP)/+*<br>• *hsFlp;UAS-LacZ-RNAi/+;Act>CD2>Gal4,UAS-nEGFP/+*<br>• *hsFlp;+/nub-Gal4;ubi-p63E>FRT.STOP.FRT>Stringer(nEGFP)/UAS-*$dlg1^{GD41136}$*-RNAi*<br>• *hsFlp;+/+;act>CD2>Gal4,UAS-nEGFP/UAS-*$dlg1^{GD41136}$*-RNAi*<br>• *hsFlp; UAS-*$NR2^{TRIP-HMS02176}$*-RNAi/nub-Gal4;ubi-p63E>FRT.STOP.FRT>Stringer(nEGFP)/+*<br>• *hsFlp;UAS-*$NR2^{TRIP-HMS02176}$*-RNAi/+;act>CD2>Gal4,UAS-nEGFP/+*<br>• *hsFlp;+/nub-Gal4;ubi-p63E>FRT.STOP.FRT>Stringer(nEGFP)/UAS-*$NR2^{GD319}$*-RNAi*<br>• *hsFlp;+/+;act>CD2>Gal4,UAS-nEGFP/*$NR2^{GD3196}$*-RNAi*
1	h-j	• *hsFlp;UAS-LacZ-RNAi/+;act>CD2>Gal4,UAS-nEGFP/+*<br>• *hsFlp;+/+;act>CD2>Gal4,UAS-nEGFP/UAS-NR2*
2	a-b	• *hsFlp;UAS-LacZ-RNAi/UAS-LacZ-RNAi;act>CD2>Gal4,UAS-nEGFP/+*<br>*hsFlp;UAS-*$NR2^{TRIP-HMS02176}$*-RNAi/UAS-LacZ-RNAi;act>CD2>Gal4,UAS-nEGFP/+*

2	c	• hsFlp;UAS-LacZ-RNAi/UAS-LacZ-RNAi;act>CD2>Gal4,UAS-nEGFP/+<br>• hsFlp;UAS-NR2$^{TRIP-HMS02176}$-RNAi/UAS-LacZ-RNAi;act>CD2>Gal4,UAS-nEGFP/+<br>• hsFlp;UAS-NR2$^{TRIP-HMS02176}$-RNAi/UAS-grnd-RNAi^{KK109939};act>CD2>Gal4,UAS-nEGFP/+<br>• hsFlp;UAS-NR2$^{TRIP-HMS02176}$-RNAi/+;act>CD2>Gal4,UAS-nEGFP/UAS-hep^{GD26929}-RNAi<br>• hsFlp;UAS-NR2^{TRIPHMS02176}-RNAi/ UAS-bsk.DN;act>CD2>Gal4,UAS-nEGFP/+<br>• hsFlp;UAS-NR2^{TRIPHMS02176}-RNAi/+;act>CD2>Gal4,UAS-nEGFP/UAS-p35,UAS-DIAP1
3	a	• hsFlp;UAS-NR2$^{TRIP-HMS02176}$-RNAi/UAS-LacZ-RNAi;act>CD2>Gal4,UAS-nEGFP/+<br>• hsFlp;UAS-LacZ-RNAi/UAS-LacZ-RNAi;act>CD2>Gal4,UAS-nEGFP/+
3	b	• hsFlp;UAS-NR2$^{TRIP-HMS02176}$-RNAi/UAS-LacZ-RNAi;act>CD2>Gal4,UAS-nEGFP/+
3	c	• hsFlp;UAS-NR2$^{TRIP-HMS02176}$-RNAi/UAS-LacZ-RNAi;act>CD2>Gal4,UAS-nEGFP/+<br>• hsFlp;UAS-LacZ-RNAi/UAS-LacZ-RNAi;act>CD2>Gal4,UAS-nEGFP/+<br>• hsFlp;UAS-NR2^{TRIPHMS02176}-RNAi/ UAS-bskDN;act>CD2>Gal4,UAS-nEGFP/+
3	d-e	• hsFlp;UAS-LacZ-RNAi/UAS-LacZ-RNAi;act>CD2>Gal4,UAS-nEGFP/+<br>• hsFlp;UAS-NR2$^{TRIP-HMS02176}$-RNAi/UAS-LacZ-RNAi;act>CD2>Gal4,UAS-nEGFP/+<br>• hsFlp;UAS-NR2$^{TRIP-HMS02176}$-RNAi/+;act>CD2>Gal4,UAS-nEGFP/Pdk-RNAi^{TRiPGL00009}<br>• hsFlp;UAS-NR2$^{TRIP-HMS02176}$-RNAi/UAS-Pdh;act>CD2>Gal4,UAS-nEGFP/+
4	b	• hsFlp; nub-Gal4, UAS-LactateFRET/tub>CD2>lexA,lexO-mCherry; lexO-LacZ/+<br>• hsFlp; nub-Gal4, UAS-LactateFRET/tub>CD2>lexA,lexO-mCherry; lexO-NR2-RNAi/+
4	c	• hsFlp;UAS-LacZ-RNAi/ UAS-LactateFRET;act>CD2>Gal4,UAS-nEGFP/+<br>• hsFlp;UAS-NR2 ^{TRIPHMS02176}-RNAi,UAS-LactateFRET/+;act>CD2>Gal4,UAS-nEGFP/+<br>• hsFlp;UAS-NR2 ^{TRIPHMS02176}-RNAi,UAS-LactateFRET/UAS-dMct1^{KK108618};act>CD2>Gal4,UAS-nEGFP/+<br>• hsFlp;UAS-LacZ-RNAi,UAS-LactateFRET/UAS-dMct1^{KK108618};act>CD2>Gal4,UAS-nEGFP/+
4	d	• hsFlp; nub-Gal4, UAS-LactateFRET/tub>CD2>lexA,lexO-mCherry; lexO-LacZ/+<br>• hsFlp; nub-Gal4, UAS-LactateFRET/tub>CD2>lexA,lexO-mCherry; lexO-NR2-RNAi/+
4	e	• hsFlp;UAS-LacZ-RNAi/ UAS-LactateFRET;act>CD2>Gal4,UAS-nEGFP/+

		• *hsFlp;UAS-NR2*^{TRIPHMS02176}*-RNAi/UAS-LactateFRET;act>CD2>Gal4,UAS-nEGFP/+*
**5**	**a**	• *hsFlp;UAS-NR2*$^{TRIP-HMS02176}$*-RNAi/UAS-LacZ-RNAi;act>CD2>Gal4,UAS-nEGFP/+*
**5**	**b**	• *hsFlp;UAS-NR2*$^{TRIP-HMS02176}$*-RNAi/+;act>CD2>Gal4,UAS-nEGFP/UAS-impL3*^{GD31192}*-RNAi*<br>• *hsFlp;UAS-NR2*$^{TRIP-HMS02176}$*-RNAi/UAS-dMct1*^{KK108618}*-RNAi;act>CD2>Gal4,UAS-nEGFP/+*<br>• *hsFlp;UAS-NR2*$^{TRIP-HMS02176}$*-RNAi/UAS-LacZ-RNAi;act>CD2>Gal4,UAS-nEGFP/+*
**5**	**c**	• *hsFlp; UAS-LacZ-RNAi/UAS-LacZ-RNAi;act>CD2>Gal4,UAS-nEGFP/+*<br>• *hsFlp;UAS-NR2*$^{TRIP-HMS02176}$*-RNAi/UAS-LacZ-RNAi;act>CD2>Gal4,UAS-nEGFP/+*<br>• *hsFlp;UAS-NR2*$^{TRIP-HMS02176}$*-RNAi/UAS-impL3*^{KK110190}*-RNAi;act>CD2>Gal4,UAS-nEGFP/+*<br>• *hsFlp;UAS-NR2*$^{TRIP-HMS02176}$*-RNAi/+;act>CD2>Gal4,UAS-nEGFP/UAS-impL3*^{GD31192}*-RNAi*<br>• *hsFlp;UAS-NR2*$^{TRIP-HMS02176}$*-RNAi/UAS-dMct1*^{KK108618}*-RNAi;act>CD2>Gal4,UAS-nEGFP/+*<br>• *hsFlp;UAS-NR2*$^{TRIP-HMS02176}$*-RNAi/UAS-LacZ-RNAi;act>CD2>Gal4,UAS-nEGFP/+*<br>• *hsFlp;UAS-NR2*$^{TRIP-HMS02176}$*-RNAi/UAS-CG13907*^{KK107339}*-RNAi;act>CD2>Gal4,UAS-nEGFP/+*<br>• *hsFlp;UAS-NR2*$^{TRIP-HMS02176}$*-RNAi/+;act>CD2>Gal4,UAS-nEGFP/UAS-CG3409*^{GD37139}*-RNAi*
**6**	**a-c,f**	• *hsFlp;UAS-LacZ-RNAi/+;act>CD2>Gal4,UAS-nEGFP/+*<br>• *hsFlp;UAS-Myc/+;act>CD2>Gal4,UAS-nEGFP/+*
**6**	**d-e**	• *hsFlp; nub-Gal4, UAS-LactateFRET/tub>CD2>lexA,lexO-mCherry;*<br>• *hsFlp; nub-Gal4, UAS-LactateFRET/tub>myc>lexA,lexO-mCherry;*
**7**	**a-e**	• *hsFlp;UAS-LacZ-RNAi/UAS-LacZ-RNAi;act>CD2>Gal4,UAS-nEGFP/+*<br>• *hsFlp;UAS-Myc/UAS-LacZ-RNAi;act>CD2>Gal4,UAS-nEGFP/+*
**7**	**f-g**	• *NR2-Gal4; UAS-GFP*
**8**	**a**	• *hsFlp;UAS-LacZ-RNAi/ UAS-LacZ-RNAi;act>CD2>Gal4,UAS-nEGFP/+*<br>• *hsFlp;UAS-Myc/UAS-LacZ-RNAi;act>CD2>Gal4,UAS-nEGFP/+*<br>• *hsFlp;UAS-Myc/UAS-NR2*$^{TRIP-HMS02176}$*-RNAi;act>CD2>Gal4,UAS-nEGFP/+*
**8**	**b**	• *hsFlp;UAS-LacZ-RNAi/ UAS-LacZ-RNAi;act>CD2>Gal4,UAS-nEGFP/+*<br>• *hsFlp;UAS-NR2*$^{TRIP-HMS02176}$*-RNAi/UAS-LacZ-RNAi;act>CD2>Gal4,UAS-nEGFP/+*<br>• *hsFlp;UAS-Myc/UAS-LacZ-RNAi;act>CD2>Gal4,UAS-nEGFP/+*<br>• *hsFlp;UAS-Myc/UAS-NR2*$^{TRIP-HMS02176}$*-RNAi;act>CD2>Gal4,UAS-nEGFP/+*
**8**	**c**	• *hsFlp;UAS-LacZ-RNAi/+;act>CD2>Gal4,UAS-nEGFP/+*

		• hsFlp;UAS-LacZ-RNAi/nub-Gal4;ubi-p63E>STOP>Stringer(nEGFP)/+<br>• hsFlp;+/nub-Gal4;ubi-p63E>STOP>Stringer(nEGFP)/NR2^{GD3196}-RNAi<br>• hsFlp;UAS-Myc,UAS-LacZ-RNAi/nub-Gal4;ubi-p63E>STOP>Stringer(nEGFP)/+<br>• hsFlp;UAS-Myc/nub-Gal4;ubi-p63E>STOP>Stringer(nEGFP)/NR2^{GD3196}-RNAi
**8**	**d**	• hsFlp;UAS-Myc/UAS-LacZ-RNAi;act>CD2>Gal4,UAS-nEGFP/+<br>• hsFlp;UAS-Myc/UAS-NR2$^{TRIP-HMS02176}$-RNAi;act>CD2>Gal4,UAS-nEGFP/+
**8**	**e-f**	• hsFlp; tub>CD2>Gal4/nub-lexA; UAS-GFP/lexO-RFP-RNAi<br>• hsFlp;tub>myc y+>Gal4, UAS-GFP/nub-LexA; lexO-RFP-RNAi/+<br>• hsFlp;tub>myc y+>Gal4, UAS-GFP/nub-LexA; lexO-NR2-RNAi/+
**8**	**g**	• hsFlp;UAS-Myc/LacZ-RNAi;act>CD2>Gal4,UAS-nEGFP/+
**8**	**h**	• hsFlp; LacZ-RNAi/LacZ-RNAi;act>CD2>Gal4,UAS-nEGFP/+<br>• hsFlp;UAS-Myc/LacZ-RNAi;act>CD2>Gal4,UAS-nEGFP/+<br>• hsFlp; LacZ-RNAi/nub-Gal4;ubi-p63E>STOP>Stringer(nEGFP)/+<br>• hsFlp;UAS-Myc/nub-Gal4;ubi-p63E>STOP>Stringer(nEGFP)/+

Supplementary Data Figures

**S1**	**a**	• W1118
**S1**	**b**	• NR2$^{MI09281-GFSTF.2}$;+;+
**S1**	**c**	• hsFlp;+/UAS-NR2$^{TRIP-HMS02176}$-RNAi;act>CD2>Gal4,UAS-nEGFP/+
**S2**	**a**	• hsFlp;+/+;act>CD2>Gal4,UAS-nEGFP/UAS-dlg1^{GD41136}-RNAi<br>• hsFlp;UAS-scrib$^{TRIP-GL00638}$-RNAi/+;act>CD2>Gal4,UAS-nEGFP/+
**S2**	**b**	• hsFlp;+/nub-Gal4;Ubi-p63E>STOP>Stringer(nEGFP)/UAS-dlg1^{GD41136}-RNAi
**S2**	**c**	• w1118;UAS-GFP/LacZ-RNAi;hh-Gal4/+<br>• w1118;UAS-GFP/ UAS-NR2$^{TRIP-HMS02176}$-RNAi;hh-Gal4/+
**S3**	**a,b**	• hsFlp;UAS-LacZ-RNAi/+;act<CD2<Gal4,UAS-nEGFP/+<br>• hsFlp;+/+;act>CD2>Gal4,UAS-nEGFP/NR2$^{TRIP-HMS02012}$-RNAi
**S4**	**a**	• hsFlp;UAS-NR2$^{TRIP-HMS02176}$-RNAi/UAS-LacZ-RNAi;act>CD2>Gal4,UAS-nEGFP/pucE69 (puc-LacZ)
**S4**	**b**	• hsFlp;UAS-LacZ-RNAi/UAS-LacZ-RNA;act>CD2>Gal4,UAS-nEGFP/+<br>• hsFlp;UAS-NR2$^{TRIP-HMS02176}$-RNAi/UAS-LacZ-RNAi;act>CD2>Gal4,UAS-nEGFP/+
**S5**	**a**	• hsFlp;UAS-bsk-WT;act>y>UAS-LacZ
**S5**	**b-d**	• hsFlp;UAS-LacZ-RNAi/UAS-LacZ-RNAi;act>CD2>Gal4,UAS-nEGFP/+<br>• hsFlp;UAS-NR2$^{TRIP-HMS02176}$-RNAi/UAS-LacZ-RNAi;act<CD2<Gal4,UAS-nEGFP/+

**S5**	**e**	• *hsFlp; UAS-NR2$^{TRIP-HMS02176}$-RNAi/nub-Gal4;ubi-p63E>FRT.STOP.FRT>Stringer(nEGFP)/+*
**S5**	**f**	• *hsFlp;UAS-LacZ-RNAi/UAS-LacZ-RNAi;act>CD2>Gal4,UAS-nEGFP/+*<br>• *hsFlp;UAS-NR2$^{TRIP-HMS02176}$-RNAi/+;act>CD2>Gal4,UAS-nEGFP/Pdk-*RNAi^{TRiPGL00009}
**S6**	**a-b**	• *hsFlp;UAS-NR2$^{TRIP-HMS02176}$-RNAi/*UAS-Pdh*;act>CD2>Gal4,UAS-nEGFP/+*
**S7**	**a-d**	• *hsFlp;UAS-LacZ-RNAi/+;act>CD2>Gal4,UAS-nEGFP/+*<br>*hsFlp;UAS-NR2$^{TRIP-HMS02176}$-RNAi/+;act<>CD2>Gal4,UAS-nEGFP/+*
**S7**	**e**	• *hsFlp;UAS-NR2$^{TRIP-HMS02176}$-RNAi/+;act<>CD2>Gal4,UAS-nEGFP/+*
**S8**	**a,b**	• *hsFlp;UAS-Myc/+;act>CD2>Gal4,UAS-nEGFP/+*
**S8**	**c**	• *hsFlp;UAS-Myc,UAS-LacZ-RNAi/nub-Gal4;ubi-p63E>STOP>Stringer(nEGFP)/+*
	**d,e**	• *hsFlp;UAS-Myc/+;act>CD2>Gal4,UAS-nEGFP/+*
**S8**	**f-g**	• *hsFlp; tub>myc>lexA,lexO-mCherry;*
**S9**		• *w1118;UAS-GFP/LacZ-RNAi;hh-Gal4/+*<br>• *w1118;UAS-GFP/LacZ-RNAi;hh-Gal4/+*
