# Supplementary material for: The NMDA receptor regulates competition of epithelial cells in the Drosophila wing
Source: Nat Commun. 2020 May 6;11:2228. doi: 10.1038/s41467-020-16070-6 (PMC7203100; doi:10.1038/s41467-020-16070-6)
Supplement: Supplementary file 3 — Reporting Summary [file 41467_2020_16070_MOESM3_ESM.pdf]

nature research

Corresponding author(s): Pascal Meier (Agnes Banreti)

Last updated by author(s): 2020 03 11

Reporting Summary

Nature Research wishes to improve the reproducibility of the work that we publish. This form provides structure for consistency and transparency in reporting. For further information on Nature Research policies, see Authors & Referees and the Editorial Policy Checklist .

Statistics

For all statistical analyses, confirm that the following items are present in the figure legend, table legend, main text, or Methods section.

n/a	Confirmed	
☐	☒	The exact sample size (*n*) for each experimental group/condition, given as a discrete number and unit of measurement
☐	☒	A statement on whether measurements were taken from distinct samples or whether the same sample was measured repeatedly
☐	☒	The statistical test(s) used AND whether they are one- or two-sided<br>*Only common tests should be described solely by name; describe more complex techniques in the Methods section.*
☐	☒	A description of all covariates tested
☒	☐	A description of any assumptions or corrections, such as tests of normality and adjustment for multiple comparisons
☐	☒	A full description of the statistical parameters including central tendency (e.g. means) or other basic estimates (e.g. regression coefficient) AND variation (e.g. standard deviation) or associated estimates of uncertainty (e.g. confidence intervals)
☒	☐	For null hypothesis testing, the test statistic (e.g. *F*, *t*, *r*) with confidence intervals, effect sizes, degrees of freedom and *P* value noted<br>*Give P values as exact values whenever suitable.*
☒	☐	For Bayesian analysis, information on the choice of priors and Markov chain Monte Carlo settings
☒	☐	For hierarchical and complex designs, identification of the appropriate level for tests and full reporting of outcomes
☐	☒	Estimates of effect sizes (e.g. Cohen's *d*, Pearson's *r*), indicating how they were calculated

Our web collection on statistics for biologists contains articles on many of the points above.

Software and code

Policy information about availability of computer code

Data collection	ZEISS ZEN was used for imaging in this study. For the purpose of visualization, the same level and channel adjustments were applied using ImageJ.
Data analysis	All statistical analyses were carried out using GraphPad Prism7 and GraphPad Prism8. Comparisons between two genotypes/conditions were analysed with the Mann-Whitney nonparametric two-tailed rank U-test or Pearson's correlation test. Values are presented as average ± standard deviation (S.D.), P-values from Mann-Whitney U-test (non-significant (ns): P>0.05; *: 0.05>P>0.1; **: 0.1>P>0.01; ***: 0.01>P>0.001;****: P>0.0001 and from Pearson's analysis α=0.05.

For manuscripts utilizing custom algorithms or software that are central to the research but not yet described in published literature, software must be made available to editors/reviewers. We strongly encourage code deposition in a community repository (e.g. GitHub). See the Nature Research guidelines for submitting code & software for further information.

Data

Policy information about availability of data

All manuscripts must include a data availability statement. This statement should provide the following information, where applicable:

- Accession codes, unique identifiers, or web links for publicly available datasets
- A list of figures that have associated raw data
- A description of any restrictions on data availability

All relevant data and materials are available from the authors upon reasonable request. The original data and statistical reports of the following figures are available in the respective Source Data file: Fig.1d,1f,1g,1i,2c,3a,3b,3e,4c,4d,4e,5c,6b,6c,6d,6e,6f,7e,8b,8c,8f,8h and S3,S5,S7c,S7d,S7e,S8d,S8e,S8f,S8g.

Field-specific reporting

Please select the one below that is the best fit for your research. If you are not sure, read the appropriate sections before making your selection.

- [x] Life sciences
- [ ] Behavioural & social sciences
- [ ] Ecological, evolutionary & environmental sciences

For a reference copy of the document with all sections, see nature.com/documents/nr-reporting-summary-flat.pdf

Life sciences study design

All studies must disclose on these points even when the disclosure is negative.

Sample size	The number of animals (Drosophila melanogaster) used per experiment was not a limiting factor in the study, thus no attempt were made to minimise the number of animals used per experiment. We used the maximum sample size that was possible, but at least triplicates were used for each condition and each genotype.
Data exclusions	We state that no data were excluded from the analyses.
Replication	All experiments were performed three independent times (made on different days) to ensure experimental reproducibility. All attemts at replication were successful.
Randomization	Randomization is not applicable to this study. Animals were allocated into different experimental groups based on their genotype and/or treatment conditions. Animals with the same genotype and/or treated in same way were allocated into differential experimental groups.
Blinding	The investigators were blinded to group allocation during data collection.

Reporting for specific materials, systems and methods

We require information from authors about some types of materials, experimental systems and methods used in many studies. Here, indicate whether each material, system or method listed is relevant to your study. If you are not sure if a list item applies to your research, read the appropriate section before selecting a response.

Materials & experimental systems

n/a	Involved in the study	
[ ]	[x]	Antibodies
[x]	[ ]	Eukaryotic cell lines
[x]	[ ]	Palaeontology
[ ]	[x]	Animals and other organisms
[x]	[ ]	Human research participants
[x]	[ ]	Clinical data

Methods

n/a	Involved in the study	
[x]	[ ]	ChIP-seq
[x]	[ ]	Flow cytometry
[x]	[ ]	MRI-based neuroimaging

Antibodies

Antibodies used	All antibodies used in this study are listed in the Materials and Methods section, with provided supplier names and lot numbers: The following primary antibodies were used: mouse anti-dNR2 (1:100, from Ann-Shyn Chiang DOI: 10.1038/nn2005), mouse anti-Dlg1 (1:50, 4F3, DSHB, University of Iowa, Iowa City, IA, USA), mouse anti-GFP (11814460001, Roche), mouse anti-MMP1 (1:50, 5H7B11, DSHB, University of Iowa, Iowa City, IA, USA), rabbit anti-cleaved DCP1 (Asp216) (1:200,Cell Signaling), mouse p-JNK (1:100, 9255,Cell Signaling Technology Inc., Danvers, MA, USA), PHA-555 (Phalloidin-555, A34055, Invitrogen/Molecular probes), mouse polyclonal anti-MCT1 (1:100, ab90582, Abcam), rabbit polyclonal anti-Myc (1:100, d1-717, sc-28207 Santa Cruz Biotechnology), rabbit polyclonal anti-p-PDHE1 (1:200, Pyruvate Dehydrogenase E1-alpha subunit (phospho S293), ab92696, Abcam). Fluorescent secondary antibodies (1:2000, FITC-, Cy3- and Cy5-conjugated) were obtained from Jackson Immunoresearch.
Validation	Drosophila-specific mouse anti-dNR2 was validated by Wu et al. DOI: 10.1038/nn2005. All other primary antibodies used in this study were validated by the manufacturer.

Animals and other organisms

Policy information about studies involving animals; ARRIVE guidelines recommended for reporting animal research

Laboratory animals	Drosophila melanogaster
Wild animals	N/A

Field-collected samples	N/A
Ethics oversight	N/A

Note that full information on the approval of the study protocol must also be provided in the manuscript.
